# Local and symptomatic control evaluation in palliative radiotherapy for rectal adenocarcinoma in frail, elderly, or metastatic patients: a multicentric retrospective study

**DOI:** 10.1016/j.ctro.2026.101231

**Published:** 2026-07-10

**Authors:** Eva Bisson, R. Nebbache, L. Quéro, C. Durdux, J.M. Simon, Y. Belkacemi, F. Huguet

**Affiliations:** a Service d'Oncologie Radiothérapie, Hôpital Tenon, AP-HP, Sorbonne Université, Paris, France; b Service de Cancérologie-Radiothérapie, Hôpital Saint-Louis, AP-HP, Université Paris Cité, Paris, France; c Département de radiothérapie, Hôpital Européen Georges Pompidou, AP-HP, Université Paris Cité, Paris, France; d Service d'Oncologie radiothérapie, Hôpitaux Universitaires Pitié Salpêtrière - Charles Foix, AP-HP, Paris, Sorbonne Université, France; e Service de Radiothérapie, Hôpital Henri-Mondor AP-HP, Créteil, France

**Keywords:** Rectal cancer, Frailty, Geriatrics, Radiation therapy, Palliative care

## Abstract

**Background:**

Locally advanced rectal cancer can cause severe pelvic symptoms (pain, hemorrhage, obstruction) in elderly, frail, or metastatic patients often unfit for surgery or optimal systemic therapy. The Swedish hypofractionated schedule (25 Gy in 5 fractions over 5 days) is widely used and effective in the neoadjuvant setting, but its role in palliation remains uncertain. Optimal dose and treatment duration remain to be defined. This study evaluates local and symptomatic control and **treatment-related** toxicity of different palliative irradiation schedules.

**Materials and methods:**

This retrospective study included patients treated with palliative radiotherapy for symptomatic, locally advanced, or metastatic rectal adenocarcinoma between 2010 and 2020 in five AP-HP Radiation Oncology departments. The primary endpoint was 6-month local control. Secondary endpoints were symptomatic response, overall survival, and toxicity. Survival was estimated using the Kaplan–Meier method and compared using log-rank test, categorical variables were compared using Fisher's exact test, and correlation using Spearman's coefficient.

**Results:**

Sixty-three patients were included (median age of 80 years) with predominantly locally advanced disease (86% T3-T4), and frequent metastases (54%). With a median follow-up of 13.3 months, the local control was 46% at 6 months (95% CI: 33.6–59) and 22.2% at 1 year (95% CI: 13–34.8). Local control did not differ significantly between irradiation schedules but tended to be higher for BED >37.5 Gy (66.7% vs. 33.3%; *p* = 0.2). A statistically significant improvement in symptomatic control was observed for BED >37.5 Gy (93.3% vs. 55.6%; *p* = 0.002), and for the 30 Gy in 10 fractions schedule (92.3% vs. 70%; *p* = 0.048). Symptomatic and local control were correlated (*p* < 0.001).

**Conclusions:**

Higher BED was associated with improved symptomatic control and a non-significant numerical increase in local control compared with the Swedish schedule. Dose escalation, including hypofractionated IMRT using simultaneous integrated boost warrant further evaluation.

## Highlights

• Palliative radiotherapy provides symptom relief in frail, elderly or metastatic patients.
• Higher biologically effective doses are associated with improved symptomatic control.
• Local control shows a trend towards improvement with higher BED schedules.
• Hypofractionated IMRT with simultaneous integrated boost warrants further evaluation.

## 1 Introduction

During the last decades, the treatment of locally advanced rectal cancer (LARC), defined as T3-T4 and/or N+ adenocarcinoma **of the mid or lower rectum**, has **achieved** substantial progress in local control and survival thanks to advances in surgical techniques such as total mesorectum excision (TME) and combination of neoadjuvant chemotherapy and chemoradiation [1], [2]. However, frail and elderly patients who are often diagnosed with LARC were not included in these studies. LARC can cause severe pelvic symptoms, such as hemorrhage, pain, bowel obstruction, or tenesmus, which can impair the quality of life (QOL) of these patients, often unfit for optimal systemic treatment and surgery, with a higher risk of toxicity or early treatment termination [3].

External beam radiation therapy (EBRT) is often used with palliative intent for symptom relief, with good efficacy. Hypofractionated schedules such as 25 Gy in 5 fractions and 5 days, have proven to be effective in the preoperative setting [1], [4], [5], with a better tolerance than conventional fractionated schedules [6], [7]. In preliminary results of a phase III randomized study, short course EBRT was also associated with better compliance in an elderly population [7]. However, in these studies, EBRT was used preoperatively, and no randomized phase III study has validated this short course EBRT scheme as a palliative treatment.

Two systematic reviews have assessed palliative pelvic radiotherapy for symptomatic rectal cancer. The first, published in 2014, reported an overall symptomatic response rate of approximately 75%, with marked heterogeneity in radiation schedules and limited data on durability of palliation and toxicity [8]. A more recent update published in 2025, including thirteen studies (five prospective), confirmed substantial variability in dose, fractionation, and associated treatments, with pooled symptomatic response rates of 71%. [9] Both reviews highlighted the absence of consensus regarding optimal dose and fractionation and the scarcity of tumour control outcomes in the palliative setting.

The aim of our study was to evaluate local and symptomatic control in LARC, as well as the toxicity of different palliative irradiation schedules, in the context of the lack of clear EBRT dose/fractionation recommendation in the literature.

## 2 Patients and methods

### 2.1 Study design and patients

This study was a retrospective analysis including patients treated with hypofractionated palliative radiotherapy for symptomatic, locally advanced or metastatic rectal adenocarcinoma between 2010 and 2020 in one of the five Radiation Oncology departments of Assistance Publique des Hôpitaux de Paris (AP-HP), with the support of the AP-HP Radiotherapy Research Group (GRRAP). The radiation schedule had to be 10 fractions or less. Patients with previous or concomitant chemotherapy treatment or discharge colostomy could be included. Patients with previous rectal irradiation were excluded, as well as patients who had undergone curative rectal surgery.

### 2.2 Endpoints

The primary endpoint of this study was local control (LC) at 6 months of the different palliative radiation schedules for symptomatic LARC or metastatic disease. Secondary endpoints were symptomatic efficacy, toxicity, and overall survival (OS).

### 2.3 Data assessment

Diagnosis date, pathology results, associated oncological treatments, initial symptoms assessment, treatment dates, and radiation technical parameters (radiation technique, volumes, dose per fraction, total dose, fractionation, treatment duration, etc.) were collected from patients' medical records, when available. Radiation techniques, doses, fractionation, and treated volumes were decided by the radiation oncologist in charge of the patient, based on the technical capacity of the center at the time of the treatment. The biologically effective dose (BED) was calculated retrospectively using the linear quadratic model with an α/β of 10 for the dose received by the tumour and with an α/β ratio of 3 for the dose received by healthy tissues. The cut-off of 37.5 Gy corresponds to the BED delivered by the Swedish schedule (25 Gy in 5 fractions, α/β = 10), which was used as the reference regimen. Tumour response and local progression were assessed by clinical examination and/or imaging, according to physician documentation. Toxicity was assessed according to the 5th version of the Common Terminology Criteria for Adverse Events (CTCAE v5).

### 2.4 Statistical analysis

The survival analyses were performed using the Kaplan-Meier method with censored survival curves at 12 months and Log-Rank calculation. Categorical variables were analyzed using Fisher's exact test, given the small sample sizes and low expected counts. Correlation between symptomatic response and tumour response was assessed using Spearman's rank correlation coefficient.

In addition, an exploratory competing-risk analysis was performed, estimating the cumulative incidence of local progression with death without prior local progression considered as a competing event.

All analyses were exploratory, *p*-values were interpreted descriptively in the context of multiple comparisons, with a two-sided p-value <0.05 considered statistically significant. Statistical analyses were performed using R software (version 4.4.1).

### 2.5 Ethics

An ethical committee's authorization was not mandatory for this retrospective analysis of already collected data, in consistency with French regulations.

## 3 Results

### 3.1 Patients' characteristics

Between January 2010 and December 2020, 707 patients were treated using hypofractionated EBRT for a LARC in the five radiation therapy departments. Sixty-three patients met our inclusion criteria (Table 1). The population was predominantly male, representing 67% of the patients with a median age of 80 years. Adenocarcinomas were mainly located in the lower (62%) and mid rectum (33%). Most tumours were considered locally advanced, with T3 in 67% of patients, 19% T4, and 68% with locoregional lymph node involvement. More than half of the patients had synchronous distant metastases (54%), mainly in the liver (38%).

**Table 1:** Patients' characteristics.

Characteristics	Total *n* = 63
**Age** (years)	
Median (range)	80 (28–95)
**Sex** (n; %)	
Man	42 (67)
Woman	21 (33)
**Location** (n; %)	
Lower rectum	39 (62)
Mid rectum	21 (33)
Upper rectum	3 (5)
**Differentiation** (n; %)	
Well	27 (43)
Moderately	27 (43)
Poorly	9 (14)
**Mutation** (n; %)	
BRAF	13 (21)
KRAS	10 (16)
HER2	1 (2)
No mutation	11 (17)
Unknown	28 (44)
**Stage T** (n; %)	
T1-T2	8 (14)
T3-T4	54 (86)
**Stage N** (n; %)	
N0	20 (32)
N+	43 (68)
**Stage M** (n; %)	
M0	29 (46)
M1	34 (54)
**Metastasis location** (n; % amon M1)	
Liver	24 (71)
Lungs	6 (18)
Others	3 (9)
**ECOG PS** (n; %)	
< 2	32 (51)
≥ 2	31 (49)
**Charlson Index** (n; %)	
< 5	25 (38)
≥ 5	40 (64)
**Oncodage G8 score** (n; %)	
> 14	4 (7)
≤ 14	43 (68)
Not Relevant	16 (25)
**Oncogeriatric assessment** (n; %)	
Yes	26 (60)
No	17 (40)
**Symptoms (**n; %)	
Rectal Bleeding	46 (73)
Rectal dysfunction	32 (51)
Pain	36 (57)
**Opioid analgesia** (n; %)	
Yes	11 (31)
No	25 (69)

Abbreviations: n, number of patients; PS, performance status.

Half of the studied population presented a poor performance status, with 49% patients having an ECOG Performance Status (PS) score ≥ 2. Most patients had multiple comorbidities, with a calculated Charlson index >5 for 64% of them. Of the 48 patients eligible for assessment using the G8 questionnaire, 43 presented potential frailty, with a G8 score < 14, representing 68% of the overall population. Of these 43 potentially frail patients, 26 underwent an oncogeriatric assessment.

The most common symptom was rectal bleeding, reported by 73% of patients. A rectal dysfunction, defined by tenesmus, false urges, and anorectal heaviness, was present in 51% of patients. Pain was also a frequent symptom, reported by more than half of the patients (57%), requiring opioid analgesic treatment for 69% of them. (Table 2).

**Table 2:** Treatments' characteristics.

Characteristics	Total n = 63
**Previous chemotherapy (n; %)**	
Yes	25 (40)
No	38 (60)
**Chemotherapy (n, %)**	
FOLFOX	9 (36)
FOLFOX Bevacizumab	6 (24)
FOLFOX Panitumumab	1 (4)
FOLFIRINOX	6 (24)
FOLFIRI Cetuximab	1 (4)
other	2 (8)
**Number of lines (n)**	
Mean (range)	1.67 (1–5)
**Discharge colostomy (n; %)**	
Yes	9 (14)
No	54 (86)
**Previous irradiation (n; %)**	
Yes	0
No	63 (100)
**Radiation schedule (n; %)**	
25 Gy / 5 f	39 (62)
30 Gy / 10 f	15 (24)
20 Gy / 5 f	3 (5)
8 Gy / 1 f	1 (2)
other	5 (10)
**Boost T (n; %)**	
Yes	2 (3)
No	61 (97)
**Radiation technique (n, %)**	
3DCRT	63 (100)
IMRT	0
**Target volume (n, %)**	
GTV T	5 (8)
GTV T + Pelvis	58 (92)
**Completed treatment (n, %)**	
Yes	61 (97)
No	2 (3)
**Concomitant chemotherapy (n)**	
Yes	9 (14)
No	54 (86)
**BED (α/β** **=** **3) (n)**	
Mean	62.96
Median	66.67
Range	29.33–96
**BED (α/β** **=** **10) (n)**	
Mean	36.63
Median	37.5
Range	14.4–62.4

Abbreviations: n, number of patients; f, fraction; 3DCRT, 3-dimensional conformal radiation therapy; IMRT, intensity modulated radiation therapy; GTV, Gross Tumour Volume; T, tumour; BED, Biologically Effective Dose.

### 3.2 Treatments characteristics

Among the included patients, 40% underwent before EBRT at least one regimen of chemotherapy, up to five. They had received an average of one line of treatment, mainly FOLFOX, either alone or in combination with bevacizumab or panitumumab (64% in total). The second most common regimen was FOLFIRINOX, used in 24% of patients. Nine patients underwent a colostomy prior to radiotherapy (14%). No patients had previously been treated with radiotherapy.

Nine different radiation schedules were identified. The most common was the Swedish regimen of 25 Gy in 5 fractions and 5 days, used in 39 patients (62%). The second most common schedule was 30 Gy in 10 fractions and 12 days in 15 patients (24%). The third most common schedule was 20 Gy in five fractions and 5 days, used in three patients (5%). The remaining five schedules were each used in one patient (2%) (Table 2).

### 3.3 Follow up

The median time to evaluation was 33 days (11−122). For assessment, 74% of patients had clinical examination, 13% CT scans, and 11% MRI. Six percent of the population did not undergo assessment after treatment. The median follow-up was13.3 months (0.5 to 59.1 months) (Table 3).

**Table 3:** Symptom response.

Characteristics	Total *n* = 62
**Time to evaluation (days)**	
Median (range)	33 (11–122)
**Type of evaluation (n; %)**	
Clinical exam	46 (74)
CT	8 (13)
MRI	7 (11)
Rectosigmoidoscopy	1 (2)
Unknown	4 (6)
**Tumoral Response (n; %)**	
CR	3 (5)
PR	19 (31)
SD	35 (56)
PD	5 (8)
**Symptom improvement (n; %)**	
Yes	47 (75)
No	15 (25)
**Rectal Bleeding**	
Resolved	28 (60)
Improved	9 (19)
Unchanged	6 (13)
Worsened	4 (8)
**Pain**	
Resolved	5 (14)
Improved	22 (61)
Unchanged	3 (8)
Worsened	6 (17)
**Rectal dysfunction**	
Resolved	6 (18)
Improved	15 (44)
Unchanged	8 (23)
Worsened	5 (15)
**Others**	
Resolved	1 (11)
Improved	2 (22)
Unchanged	2 (22)
Worsened	4 (45)
**Progression (n; %)**	
Yes	45 (71)
No	15 (24)
Unknown	3 (5)
**Progression location (n; %)**	
Local	25 (56)
Distant	20 (44)
**Time to local progression (months)**	
Median (range)	4,1 (0,7-44,8)

Abbreviations: n, number of patients; CT, computed tomography; MRI, magnetic resonance imaging; CR, complete response; PR, partial response; SD, stable disease; PD, progressive disease.

### 3.4 Local control

At the first assessment, 56% of patients had stable tumours. A partial response was observed in 31% of patients, a complete response in 5%, and a disease progression in 8%.

Forty-five patients (71%) presented a progression during the follow-up. Among them, 56% experienced local progression and 44% developed new or progressive metastatic disease. The median time to local progression was 4.1 months. Among patients with local progression, 80% happened within 6 months, and 92% within one year (Table 3).

**Fig. 1:**
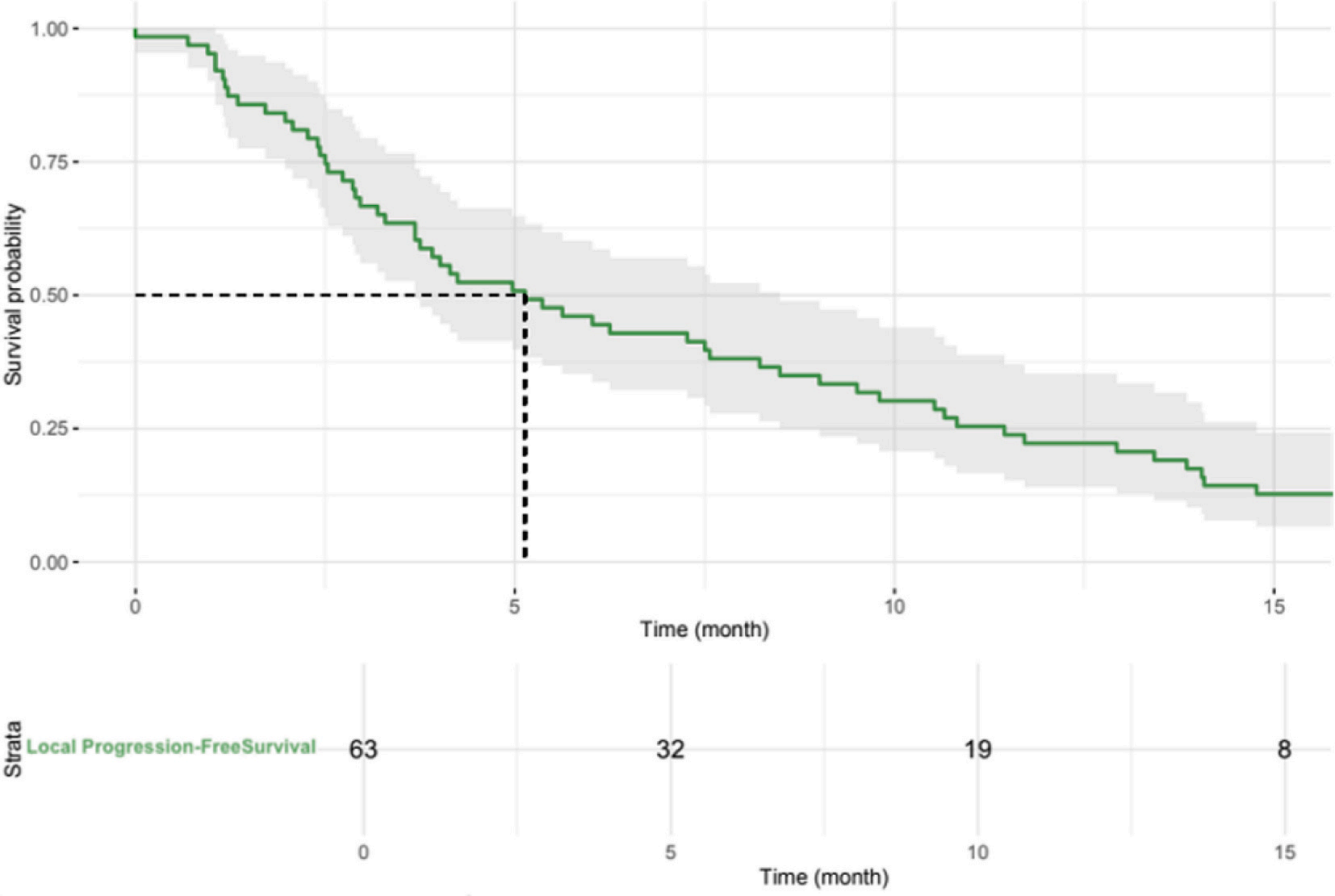
Kaplan-Meier curve for local progression-free survival. Median local progression-free survival was 5.4 months.

The progression-free survival rate was 31.7% (95% CI: 21%–45%) at 6 months and 15.9% (95% CI: 8.2%–27.7%) at 12 months. The local progression-free survival rate was 46% (95% CI: 33.6%–59%) at 6 months and 22.2% (95% CI: 13%–34.8%) at 1 year (Fig. 1). The median survival time without local progression was 5.4 months.

In univariate analyses, there was no statistically significant association between local control rates and the parameters studied, including age, TNM stage, ECOG PS, radiation regimen, or BED (Table 4).

**Table 4:** Univariate analysis of local control and symptomatic control.

**Parameter (n, %)**	**Local control**	**p**	**Symptomatic control**	**p**
**Age (years)**				
< 75	11 (55%)	0.5968	16 (80%)	0.7187
≥ 75	19 (44.2%)		31 (72.1%)	
**Stage T**				
≤ 2	5 (63.5%)	0.6009	6 (75%)	1
> 2	25 (45.5%)		41 (74.5%)	
**Stage N**				
N0	8 (44.4%)	0.9682	11 (61.1%)	0.2166
N+	22 (48.9%)		36 (80%)	
**Stage M**				
M0	15 (48.2%)	1	20 (69%)	0.51
M1	15 (47.1%)		27 (79.4%)	
**ECOG PS**				
< 2	15 (46.9%)	1	23 (71.8%)	0.829
≥ 2	15 (48.4%)		24 (77.4%)	
**BED α/β** **=** **10**				
< 37.5	3 (33.3%)	0.2047	5 (55.6%)	**0.002711**
37.5	17 (43.6%)		28 (71.8%)	
> 37.5	10 (66.7%)		14 (93.3%)	
**Radiation schedule**				
8 Gy / 1 f	0 (0%)	0.280	0 (0%)	**0.04836**
25 Gy / 5 f	17 (44.3%)		28 (71.8%)	
30 Gy / 10 f	9 (69.2%)		12 (92.3%)	
Other	4 (40%)		7 (70%)	

Abbreviations: n, number of patients; PS, performance status; BED, Biologically Effective Dose; f, fraction.

### 3.5 Symptoms palliation

Seventy-five percent of patients experienced an overall improvement in symptoms after treatment. Regarding rectal bleeding, pain, and rectal dysfunction, symptom resolution or improvement was observed respectively in 79%, 75%, and 62% of patients, and symptom stability in 13%, 8%, and 23% of patients. However, fatigue worsened after treatment in 45% of patients (Table 3).

In univariate analyses, there was a statistically significant association between symptomatic response, radiation schedule, and BED. Symptom improvement was higher for BED >37.5 Gy (93%) compared with BED = 37.5 (71.8%) and BED <37.5 (55.6%) (*p* = 0.0027). This improvement was more pronounced in patients treated with the 30 Gy in 10 fractions regimen, 92.3% versus 71.8% for the Swedish regimen, and 70% for the other regimens (*p* = 0.04836). These results suggested greater symptomatic efficacy of treatments with the highest BED. (Table 4).

After correlation analysis, a statistically significant association was found between symptoms regression and tumour response (ρ = −0.491, *p* < 0.001), indicating that better tumour response was associated with greater symptoms regression.

### 3.6 Overall survival

**Fig. 2:**
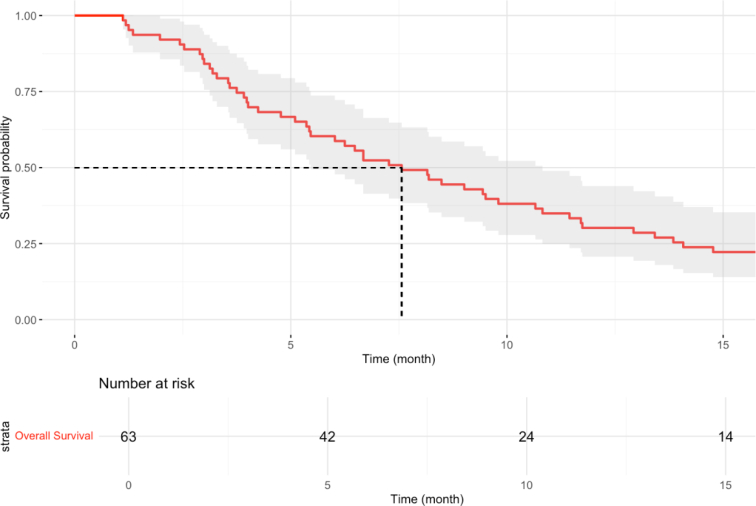
Kaplan-Meier curve for overall survival. Median overall survival was 7.6 months.

At the time of data collection, only one patient was alive. Six-month overall survival rate was 60% (95% CI: 49.4%–73.7%) and one-year overall survival rate 30.2% (95% CI: 20.7%–43.9%). Median overall survival was 7.57 months (Fig. 2).

### 3.7 Competing risk analysis

The cumulative incidence of local progression, considering death without prior local progression as a competing event, was 33.3% at 6 months and 38.1% at 12 months. The cumulative incidence of death without prior local progression was 20.6% at 6 months and 39.7% at 12 months, indicating that a substantial proportion of patients died before experiencing local progression.

### 3.8 Toxicity

During treatment, 82% of patients experienced at least one acute toxicity. Most of these toxicities were gastrointestinal (73%), mainly grade 1 or 2 diarrhea, rectal bleeding, or anitis. Three grade 3 acute gastrointestinal toxicities were reported in 6% of patients, respectively one episode of diarrhea requiring hospitalization, one rectal bleeding requiring transfusion, and one episode of anitis. Few acute urological toxicities were reported, mainly grade 1 or 2 pollakiuria or dysuria (16%). Genital toxicities were rare, found in 6% of patients, mainly grade 1 or 2 vulvitis. No acute urinary or genital toxicities of grade 3 or higher were reported.

Data on late toxicity (more than 3 months after the end of the treatment) were available for 96% of the studied cohort. Only 25% of patients experienced grade 1 or 2 late toxicity. No grade 3 or higher late toxicity was reported. Late toxicities were mainly digestive, with 14% of patients experiencing grade 1 or 2 diarrhea or anal incontinence. Late urological toxicity was found in 10% of patients, with grade 1 or 2 pollakiuria or incontinence. (Table 5)

**Table 5:** Acute and late toxicities.

Adverse Event (n;%)	Acute Toxicity	Late Toxicity
Gastrointestinal	37 (72)	8 (53)
Grade 1	18 (35)	5 (33)
Grade 2	16 (31)	3 (20)
Grade 3	3 (6)	0
Grade 4	0	0
Urinary	10 (20)	6 (40)
Grade 1	7 (14)	4 (27)
Grade 2	3 (6)	2 (13)
Grade 3	0	0
Grade 4	0	0
Genital	4 (8)	1 (7)
Grade 1	2 (4)	0
Grade 2	2 (4)	1 (7)
Grade 3	0	0
Grade 4	0	0

After univariate analysis, there was no statistically significant association between acute toxicity and radiation modalities, BED, and chemotherapy.

## 4 Discussion

In the palliative setting, symptoms relief remains the primary clinical objective. In this context, the present study sought to examine whether differences in tumour response and LC between commonly used hypofractionated radiotherapy schedules could be associated with variations in the durability and magnitude of symptomatic benefit in frail, elderly, or metastatic patients. To the best of our knowledge, this study is the first to evaluate LC of different palliative radiation schedules in this context. Most published studies have evaluated symptomatic response, while limited data exists on tumour response, its predictive factors, and its correlation with symptomatic response. In a retrospective setting, tumour response and progression are generally more consistently documented and comparable across centres than symptom outcomes, which are inherently subjective and may be influenced by supportive care interventions. Symptomatic control was therefore analyzed as a key secondary endpoint and interpreted in conjunction with tumour response.

Data on tumour control outcomes in palliative rectal radiotherapy remain scarce. Only two retrospective studies have reported local control or survival as primary or secondary endpoints. [10], [11] Jain et al. reported a median overall survival of 12 months in patients treated with short-course radiotherapy combined with chemotherapy [10], while Hoskin et al. described high response rates of 84% using intracavitary brachytherapy in a small heterogeneous cohort. Differences in assessment methods, radiation dose, and patient selection limit direct comparisons with our results. Although a numerical difference in terms of BED was observed in our univariate analysis, with a 6-month LC estimated at 33.3% for BED <37.5, and 66.7% for BED >37.5, this difference did not reach statistical significance (*p* = 0.2047). This non-significant difference was also observed for the different radiation regimens, in favor of the 30 Gy/10f schedule with a 6-month LC of 69.2% compared to 43.6% for the Swedish schedule and 40% for the others (*p* = 0.2804). This lack of statistical significance can be explained by the small size of the population studied and the significant heterogeneity of the other schedules group, which included doses ranging from 15 Gy in 3 fractions to 36 Gy in 12 fractions. Even if the Swedish regimen was the most widely used in our study, it does not appear to offer the most optimal LC rate. This result is consistent with the low complete response rate found in operative specimens treated with the Swedish schedule alone, estimated at 0.7% in the study by Bujko et al. [6].

Our study shows in univariate analyses an improvement in symptomatic control for higher BED as well as for schedules of at least 30 Gy in 10 fractions compared to the other studied. Indeed, a better symptomatic control was found for BED above 37.5 Gy, 93.3% versus 55.6% for BED under 37.5 Gy (*p* = 0.0027). This improvement was also greater in patients treated with the 30 Gy in 10 fractions schedule (BED = 39) at 92.3%, versus 71.8% for the Swedish schedule (BED = 37.5), and 70% for the others (*p* = 0.048). Acute toxicity did not appear to be increased for higher doses and remained acceptable, with only 6% of grade 3 gastrointestinal toxicities and a majority of grade 1 or 2 toxicities. The correlation between significant symptomatic improvement and local control (ρ = − 0.491, *p* < 0.001) also supports the trend of improved local control with higher doses. While symptomatic relief can be achieved transiently through supportive measures, control of local tumour progression may play an important role in maintaining longer-term symptom improvement, particularly for bleeding, pain, and rectal dysfunction.

However, these results should be interpreted with caution. Treatment selection was clinician-dependent, and indication bias cannot be excluded, as dose and fractionation may have been influenced by baseline clinical status, estimated life expectancy or metastatic burden. In addition, multivariable analyses were not feasible given the limited sample size and number of events, particularly for local progression and non-response outcomes, with a high risk of model overfitting and unstable estimates. Therefore, the observed associations between BED, symptomatic control, and local control should be interpreted with caution, as they may partly reflect confounding related to performance status, metastatic burden, frailty, or clinician-driven treatment selection.

Palliative radiation therapy also appears to be beneficial for metastatic patients, as reported in several other retrospective studies [10], [12], [13], [14]. Our results are consistent with those in literature; with an overall symptoms improvement of 75%, compared to 71% in our recent systematic review, and 75% in 2014 review by Cameron et al. However, specific response rates for pain, rectal bleeding, and rectal dysfunction, at 75%, 79% and 62% respectively, were slightly lower than those found in literature, at 90%, 85%, and 84% in our review [9], and comparable to Cameron's review, at 78%, 81%, and 71% [8]. These variations could be explained by differences in symptom assessment, associated oncological or supportive treatments, and the heterogeneity of the radiation schedules and techniques studied, which were often normofractionated, and not suitable for palliative patients.

The correlation between symptomatic control and higher dose delivered has been reported in literature, especially for hemostatic irradiation. A systematic review of 58 articles, found that a higher median dose (at least 20 Gy in their review) associated with a hypofractionated irradiation schedule, was associated with better hemostatic control [15].

This study is limited by its retrospective, multicentre design and the relatively small sample size, which precluded multivariable analyses and may have introduced indication bias, as dose and fractionation were selected at the discretion of the treating physician. Symptom assessment relied on routine clinical documentation rather than standardized patient-reported outcome measures and may have been influenced by concomitant supportive care interventions. In addition, follow-up evaluations were heterogeneous, relying predominantly on clinical examination, occasionally imaging, performed at variable time points, which may have affected the LC evaluation. This heterogeneity may have resulted in an underestimation of both tumour response and symptomatic benefit in some cases, particularly when assessments were performed too early to capture maximal treatment effect. In this frail population with limited survival, death represents a major competing event for local progression. In our cohort, the cumulative incidence of local progression at 6 months was 33.3%, while 20.6% of patients died without prior local progression. Accordingly, Kaplan-Meier estimates should be interpreted as the probability of being alive without local progression rather than a direct measure of tumour control. Finally, a subset of patients received concomitant systemic therapy during radiotherapy, including chemotherapy associated with anti VEGF agents such as bevacizumab (9%). Although anti-angiogenic agents are generally avoided during pelvic irradiation because of an increased risk of gastrointestinal toxicity, their use in this cohort reflects real-world practice and may have influenced toxicity and symptom outcomes. [16], [17], [18]. Because of the retrospective design of the study, the exact timing of systemic therapy relative to radiotherapy was not consistently available and was therefore not collected. Consequently, its potential influence on treatment-related toxicity could not be reliably assessed.

Given all these limitations, a radiation dose escalation on the tumour may improve symptomatic control and could be associated with improved local control, while maintaining acceptable toxicity. Dose escalation through integrated boost with IMRT/VMAT techniques are increasingly used in daily practice. A feasibility study published in 2023 investigated dose escalation with simultaneous integrated boost (SIB) using IMRT for various tumours larger than 4 cm, for schedules of 25 Gy in 5 fractions and SIB of 50 Gy in 5 fractions or 30 Gy in 10 fractions and SIB of 60 Gy in 10 fractions with a target dose delivery of at least 150% of the prescribed dose to the tumour [19]. Over a 42-day follow-up period, the 21 patients studied received the prescribed doses without increased toxicity or interruption of treatment. The efficacy of contact therapy irradiation could also be evaluated, as it appears to be less invasive and less toxic than brachytherapy, and allows for an interesting dose escalation for smaller tumours, as demonstrated in the OPERA trial [20].

## 5 Conclusions

Although LC rates did not differ significantly between the different palliative radiation schedules in our cohort of LARC in elderly, frail, or metastatic patients, a numerical increase in LC at 6 months was observed for BED greater than 37.5 Gy. There was also a statistically significant improvement in symptomatic control in favor of BED greater than 37.5 Gy, with the 30 Gy in 10 fractions schedule, as well as a correlation between symptomatic control and local control. These results suggest that higher BED regimens may contribute to improved symptomatic control compared to the Swedish schedule (25 Gy in 5 fractions over 5 days). Further prospective studies are needed to define the optimal dose escalation and fractionation in this setting.

## Declaration competing interest

The authors declare no competing interests.

## CRediT authorship contribution statement

EB conceived the study, coordinated data collection, and drafted the manuscript. RN performed the statistical analyses and contributed to the interpretation of the results. FH supervised the study and critically revised the manuscript. All authors approved the final manuscript.
